# Precision of Ablation Margin Assessment After Liver Microwave Ablation: A Head-to-Head Comparison of Visual and Software-Assisted Methods

**DOI:** 10.1007/s00270-026-04528-x

**Published:** 2026-07-03

**Authors:** Coline Morisot, Idriss Chami Khazraji, Vincent Habouzit, Nicolas Magand, Bertrand Le Roy, Ilies El Boukili, Claire Boutet, Sylvain Grange, Rémi Grange

**Affiliations:** 1 Department of Radiology, University Hospital of Saint-Etienne, Saint-Priest-en-Jarez, France; 2 Department of Nuclear Medicine, University Hospital of Saint-Etienne, Saint-Priest-en-Jarez, France; 3 Department of Oncologic and Digestive Surgery, University Hospital of Saint-Etienne, Saint-Priest-en-Jarez, France; 4 Department of Radiology, University Hospital of Saint-Étienne, 42055 Saint-Étienne Cedex 2, France

**Keywords:** Microwave ablation, Liver, Reproducibility, Minimal ablative margin, Hepatocellular carcinoma, Colorectal liver metastases

## Abstract

**Purpose:**

Minimal ablative margins (MAM) are critical for local tumour control after microwave ablation, yet their assessment remains subjective. We evaluated whether software improves the precision of ablation margin assessment after liver MWA.

**Materials and Methods:**

In this retrospective study, patients undergoing CT-guided liver MWA between January 2019 and January 2025 were included. Two radiologists (with 4 and 5 years of experience in abdominal imaging) assessed MAM in two steps: first by side-by-side visual comparison of pre- and post-ablation CT using anatomical landmarks, then with dedicated software providing semi-automatic 3D co-registration and margin quantification. Each method included two reading sessions per reader, six weeks apart. Precision was assessed using intraclass correlation coefficients (ICC), Cohen’s kappa (κ) for categorical classification (< 5, 5–10, > 10 mm), and Bland–Altman analysis.

**Results:**

Sixty-seven patients with 87 liver tumours were included. Visual assessment showed poor reliability, with low intrareader agreement (ICC 0.10 and 0.33) and near-random interreader agreement (κ =  − 0.10; ICC − 0.05), with limits of agreement approaching 17 mm. Software-assisted assessment improved reliability, with higher intrareader ICCs (0.54 and 0.83) and improved interreader agreement (κ = 0.38; ICC = 0.54). Interreader bias decreased from − 2.84 to + 0.91 mm, and limits of agreement narrowed to − 4.10 to + 5.92 mm (*p* < 0.001). The median absolute interreader difference decreased from 3.0 to 1.0 mm (*p* < 0.001).

**Conclusion:**

Software-assisted assessment improves reliability of MAM evaluation compared with visual methods, but interreader agreement remains only fair-to-moderate; further validation is required before routine clinical adoption.

**Graphical Abstract:**

**Figure:**
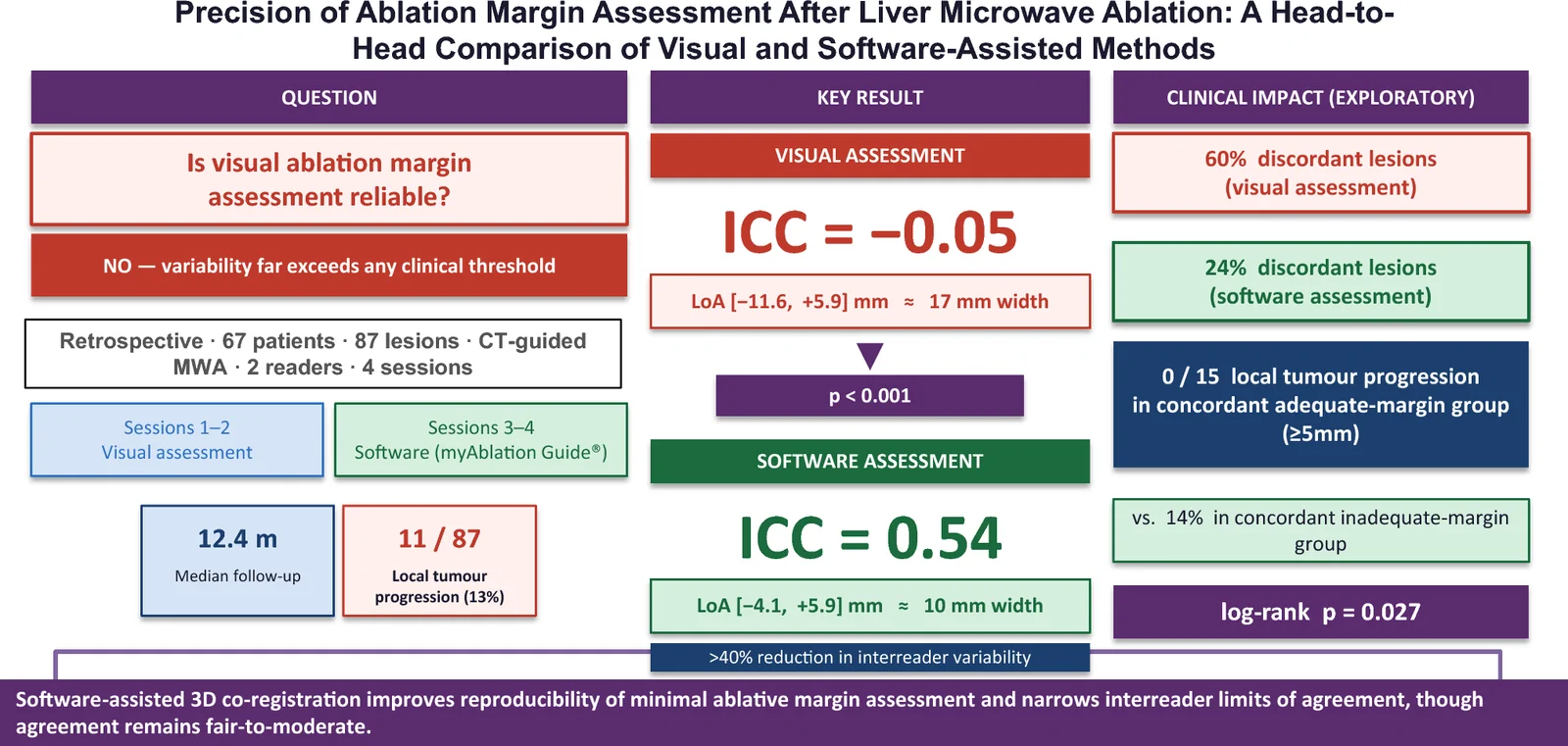

**Supplementary Information:**

The online version contains supplementary material available at https://doi.org/10.1007/s00270-026-04528-x.

## Introduction

Microwave ablation (MWA) is widely used for primary and secondary liver malignancies, with local tumour control critically dependent on adequate ablative margins. [1, 2].

Several studies have demonstrated that minimal ablative margins (MAM) ≤ 5 mm are associated with significantly higher rates of local tumour progression (LTP) [3–5]. However, accurate assessment of these minimal ablation margins remains challenging in clinical practice [6, 7]. Consequently, the reliability of visual margin assessment as a basis for clinical decision-making remains uncertain.

Despite growing interest in dedicated software tools for objective ablation margin quantification through image co-registration [8–10], their repeated-reader precision remains poorly characterised. Although software-assisted margin quantification has been shown to outperform visual evaluation, including the recent COVER-ALL randomised trial, data on intrareader repeatability and interreader reproducibility remain limited [11, 12].

Accordingly, the aim of the present study was to perform a head-to-head precision analysis with repeated blinded sessions in a cohort of patients undergoing computed tomography (CT)-guided liver MWA.

## Materials and Methods

### Population

This study was reported in accordance with the STROBE (Strengthening the Reporting of Observational Studies in Epidemiology) guidelines. All patients who underwent CT-guided liver MWA between January 2019 and January 2025 were retrospectively reviewed. Exclusion criteria included: procedures performed without contrast-enhanced imaging before and after MWA, tumours not visible on pre-ablation enhanced acquisition, and patients lost to follow-up. Patients with hepatocellular carcinoma (HCC) were considered eligible for thermal according to the Barcelona Clinic Liver Cancer staging system (BCLC-A) [2]. Patients with colorectal liver metastases (CRLM) were considered for thermal ablation when surgery was not feasible and all metastatic sites could be treated with curative intent. Tumour size was considered as one factor in treatment allocation rather than a strict cut-off. This study was conducted in accordance with the Declaration of Helsinki and received approval from the institutional ethics committee (IRB 2025-173).

### Procedure

All cases had been discussed and validated in a multidisciplinary tumour board including at least an interventional radiologist, a hepatobiliary surgeon and a medical oncologist. All procedures were performed under general anaesthesia under CT guidance (Somatom Confidence 20, Siemens Healthineers, Erlangen, Germany). Acquisitions were obtained during a controlled end-expiratory apnoea, so that pre- and post-ablation datasets were acquired in a comparable respiratory phase. Pre-procedural CT included a venous phase at 80 s for CRLM, and a triphasic protocol (unenhanced, arterial at 45 s, venous at 80 s) for HCC and cholangiocarcinoma. All pre- and post-procedural acquisitions were reconstructed with a 2 mm slice thickness. Antenna placement was performed under CT or combined CT and ultrasound guidance, at the operator’s discretion. All procedures were conducted by four radiologists with 5–10 years of experience in hepatic ablation. A single microwave generator (Emprint, Covidien, Medtronic, Dublin, Ireland) was used for all procedures. In accordance with the CIRSE Standards of Practice on Thermal Ablation of Liver Tumours, the intended minimum tumour-free ablation margin was 5 mm [6]. Margins were visually evaluated directly after the ablation with the aim of performing a complementary overlapping ablation where necessary. Hydrodissection with sterile glucose 5% was performed at the operator’s discretion.

Post-ablation imaging was systematically performed with at least a venous phase to assess ablation margins and detect potential complications. Follow-up imaging was performed at 1, 3, 6 and 12 months, then every 6 months up to year 3 and yearly thereafter.

### Assessment of Minimal Ablation Margins

Before reader assessments, eligibility was verified by a senior radiologist (RG, 10 years of experience in gastrointestinal imaging), who confirmed availability of pre- and post-ablation contrast-enhanced CT and unambiguous tumour visibility. This reviewer was not involved in subsequent reliability sessions.

**Fig. 1:**
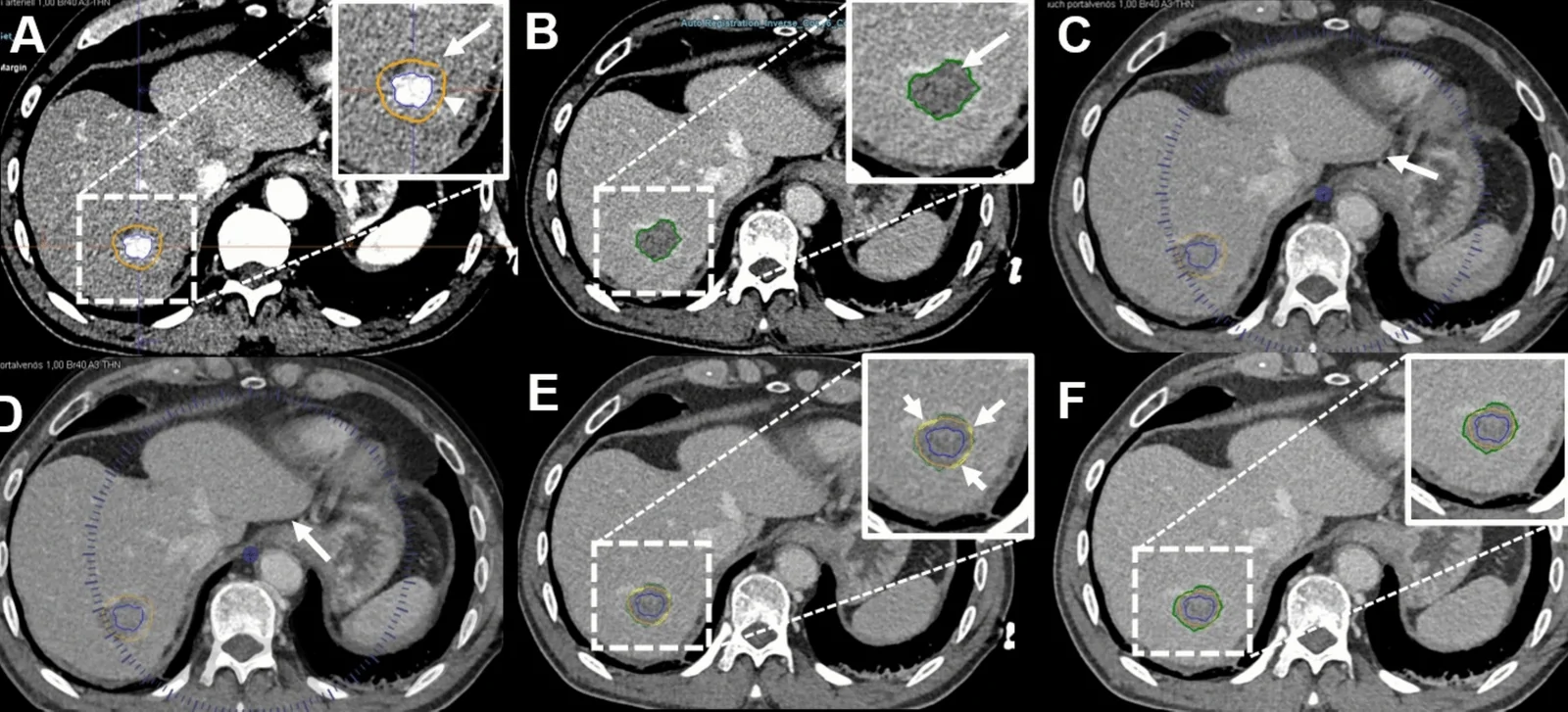
Semi-automated workflow for minimal ablative margin (MAM) assessment. **A** Pre-ablation contrast-enhanced CT (arterial phase): semi-automatic segmentation of the target tumour (white arrow) and application of a 4 mm reference margin (arrowhead). **B** Post-ablation contrast-enhanced CT (venous phase): segmentation of the ablation zone (white arrow). **C** Initial automatic rigid co-registration of the pre- and post-ablation datasets, showing suboptimal alignment along the posterior aspect of the left hepatic lobe (white arrow). **D** After manual refinement of the registration, alignment of the posterior aspect of the left hepatic lobe is satisfactory (white arrow). **E** Resulting margin map: incomplete coverage of the 4 mm reference margin is identified at three sites displayed as yellow lines (arrows), indicating an inadequate ablative margin at this threshold. **F** Same lesion with a 3 mm reference margin tested: complete coverage is achieved, illustrating the stepwise procedure used to determine the recorded MAM: MAM was recorded as 3 mm (Courtesy of University Hospital Erlangen, Prof. Matthias May) MAM, minimal ablative margin; CT, computed tomography

Ablation margin assessment was performed in two sequential steps by two radiologists (ICK and CM, 4 and 5 years of experience in gastrointestinal imaging, and 2 and 3 years of experience in liver ablation, respectively), working independently and blinded to each other’s measurements throughout the study. In the first step, margins were assessed visually by side-by-side comparison of pre- and post-ablation CT images, without software co-registration, PACS-based image fusion, or hybrid visual-registration workflows, as this is the approach routinely used in our practice. Each reader identified the shortest distance between the tumour boundary on pre-ablation imaging and the outer edge of the ablation zone on post-ablation imaging, using anatomical landmarks (vessels, bile ducts, liver contour) for spatial alignment. The minimal ablative margin (MAM) was defined as this shortest tumour-to-ablation zone distance, measured in millimetres along the most critical axis. Each reader performed two independent assessment sessions separated by a six-week interval to minimise recall bias (Session 1 and 2). In the second step, the same datasets were re-evaluated using dedicated software (myAblationGuide, Siemens Healthineers). For each lesion, the target tumour and the ablation zone were segmented semi-automatically: the software generated an initial automatic segmentation on the pre-ablation and post-ablation contrast-enhanced CT, respectively, which each reader independently reviewed and manually adjusted when judged suboptimal. The software then performed automatic rigid co-registration of the two datasets, which each reader could likewise manually refine. Rather than directly measuring a tumour-to-ablation-zone distance, the reader tested successive candidate values of the required MAM: for a given value N, the software determined whether the ablation zone fully encompassed the tumour expanded by an N-mm margin. The reader incremented this value stepwise; the minimal ablative margin recorded for the lesion was the largest value N for which complete coverage was still confirmed, the next increment (N + 1) no longer being satisfied (Fig. 1). Each reader performed two independent blinded sessions separated by a six-week interval to minimise recall bias (Session 3 and 4), following a standardised training phase of ten cases.

### Patient Data

Clinical variables, procedural characteristics, and oncological data were collected from electronic medical records. Patient outcomes, including local tumour progression (LTP), distant intrahepatic progression, distant extrahepatic progression, and overall survival (OS), were recorded. Adverse events were recorded according to modified CIRSE classification [13].

LTP was defined as the appearance of tumour foci at or adjacent to the ablation zone, whereas distant intrahepatic progression was defined as the appearance of new non-contiguous hepatic tumour. Corresponding progression-free survival endpoints (LTPFS) and distant intrahepatic progression-free survival (PFS) were defined as time from ablation to event or last imaging follow-up. OS was defined as the time from ablation to death from any cause or last follow-up. OS and LTPFS were estimated using the Kaplan–Meier method.

### Statistical Analysis

The primary endpoints were intrareader repeatability and interreader reproducibility of MAM assessment for visual and software-assisted methods. The exploratory endpoint was the association between interreader margin concordance and LTPFS.

Each radiologist performed four reading sessions: two visual (sessions 1–2), and two software-assisted (sessions 3–4) separated by 6 weeks. Following Quantitative Imaging Biomarkers Alliance (QIBA) terminology, repeatability (intrareader) was estimated from sessions 1 versus 2 (visual) and 3 versus 4 (software); reproducibility (interreader) from session 1 (visual) and session 3 (software) [14].

Patient and tumour characteristics were summarised as counts and percentages or medians with interquartile ranges (IQR). Continuous MAM agreement was quantified by intraclass correlation coefficients (ICC, two-way mixed-effects, absolute agreement) with 95% confidence intervals, interpreted as poor (< 0.50), moderate (0.50–0.75), good (0.75–0.90) and excellent (> 0.90). Categorical agreement, after classifying MAM as < 5 mm, 5–10 mm or > 10 mm, was assessed by Cohen’s weighted κ, interpreted per Landis and Koch as poor (< 0.00), slight (0.00–0.20), fair (0.21–0.40), moderate (0.41–0.60), substantial (0.61–0.80) and almost perfect (0.81–1.00) [15–17]. Bland–Altman analysis was used to estimate systematic bias and 95% limits of agreement (LoA = bias ± 1.96 standard deviation (SD)). Mean bias was compared with a paired t test, LoA width with Pitman’s test, and median absolute differences with the Wilcoxon signed-rank test. OS and LTPFS were estimated by the Kaplan–Meier method.

For the exploratory analysis, tumours were classified as concordant inadequate, concordant adequate, or discordant according to the 5 mm threshold. LTPFS was compared using the log-rank test. Statistical significance was set at *p* < 0.05. Analyses were performed using R (version 4.4.1).

## Results

### Population

**Fig. 2:**
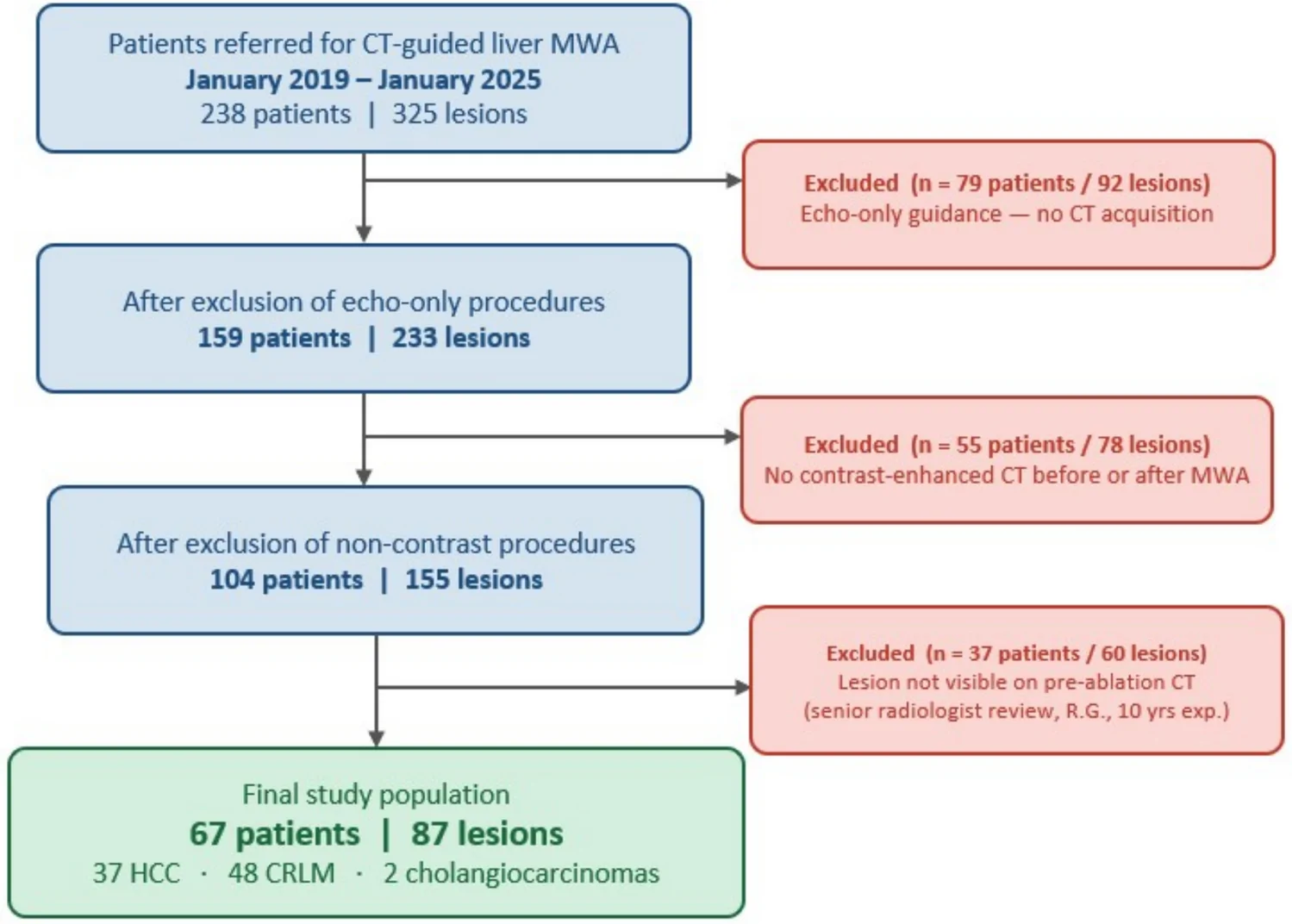
Flow chart of the study population (n = 67)

From January 2019 to January 2025, 238 patients referred for CT-guided liver MWA were retrospectively reviewed. Sixty-seven patients with 87 tumours treated during 73 procedures were included in the final analysis (Fig. 2). The cohort comprised 37 (43%) HCC, 48 (55%) CRLM and 2 (2%) cholangiocarcinomas. Median age was 70 [IQR 60–77] years, and 54 (81%) patients were male (Table 1).

**Table 1:** Patient, tumour, procedural and oncological characteristics

Characteristic	Value
*Patient characteristics (n* = *67 patients)*	
Age, years, median [IQR]	70 [60–77]
Male sex, n (%)	54 (81%)
ASA score, median [IQR]	3 [2, 3]
ASA 2, n (%)	28 (42%)
ASA 3, n (%)	38 (57%)
ASA 4, n (%)	1 (1%)
Diabetes mellitus, n (%)	21 (31%)
Cardiac disease, n (%)	30 (45%)
Prior hepatic surgery, n (%)	24 (36%)
Prior thermal ablation, n (%)	18 (27%)
*Tumour characteristics (n* = *87 tumours)*	
Tumour type	
HCC, n (%)	37 (43%)
CRLM, n (%)	48 (55%)
Cholangiocarcinoma, n (%)	2 (2%)
Tumour size, mm, median [IQR]	17 [12–23]
HCC, mm, median [IQR]	23 [18–27]
CRLM, mm, median [IQR]	14 [9–17]
Distance to capsule, mm, median [IQR]	5 [0–20]
Subcapsular location (distance = 0 mm), n (%)	34 (39%)
*Number of tumours per patient*	
Median (range)	1 (1–3)
1 tumour, n (%)	61 (84%)
2 tumours, n (%)	10 (14%)
3 tumours, n (%)	2 (3%)
*Lesion location by hepatic segment*	
Segment I, n (%)	1 (1%)
Segment II, n (%)	8 (9%)
Segment III, n (%)	9 (10%)
Segment IV, n (%)	6 (7%)
Segment V, n (%)	10 (11%)
Segment VI, n (%)	16 (18%)
Segment VII, n (%)	20 (23%)
Segment VIII, n (%)	17 (20%)
Left liver (segments I–IV), n (%)	24 (28%)
Right liver (segments V–VIII), n (%)	63 (72%)
*Procedural characteristics (n* = *73)*	
Image guidance	
CT guidance only, n (%)	38 (52%)
CT + ultrasound guidance, n (%)	35 (48%)
Number of ablations per lesion, median [IQR] (range)	1 [1, 2] (1–5)
Microwave power, W, median [IQR]	100 [85–100]
Ablation duration per lesion, min, median [IQR]	10 [8–13]
Hydrodissection, n (%)	15 (21%)
Procedure duration, min, median [IQR]	62 [46–86]
Length of hospitalisation, days, median [IQR]	1 [1–1]
Adverse events grade ≥ 3 (modified CIRSE classification), n (%)	0 (0%)
*Follow-up and oncological outcomes*	
Hepatic imaging follow-up, months, median [IQR]	12.4 [5.7–24.6]
Deaths during follow-up, n (%)	15/67 (22%)
Overall survival	
1-year OS, %	94.1%
2-year OS, %	88.5%
3-year OS, %	83.3%
Local tumour progression (LTP), n (%)	11/87 (13%)
Local tumour progression-free survival	
1-year LTPFS, %	92.9%
2-year LTPFS, %	85.7%
3-year LTPFS, %	71.6%
Distant intrahepatic progression, n (%)	36/67 (54%)
Extrahepatic progression, n (%)	33/67 (49%)

Data are presented as n (%) for categorical variables and median [interquartile range] for continuous variables. Survival estimates were derived using the Kaplan–Meier method. IQR, interquartile range; HCC, hepatocellular carcinoma; CRLM, colorectal liver metastases; ASA, American Society of Anaesthesiologists physical status classification; OS, overall survival; LTPFS, local tumour progression-free survival; W, watts

### Procedure

The median number of tumours treated per session was 1 (range: 1–3), with a median procedure duration of 62 [IQR 46–65] min and a median length of hospitalisation of 1 [1-1] day. No adverse event graded ≥ 3 (modified CIRSE classification) was recorded (Table 1).

### Evaluation of Margins

**Fig. 3:**
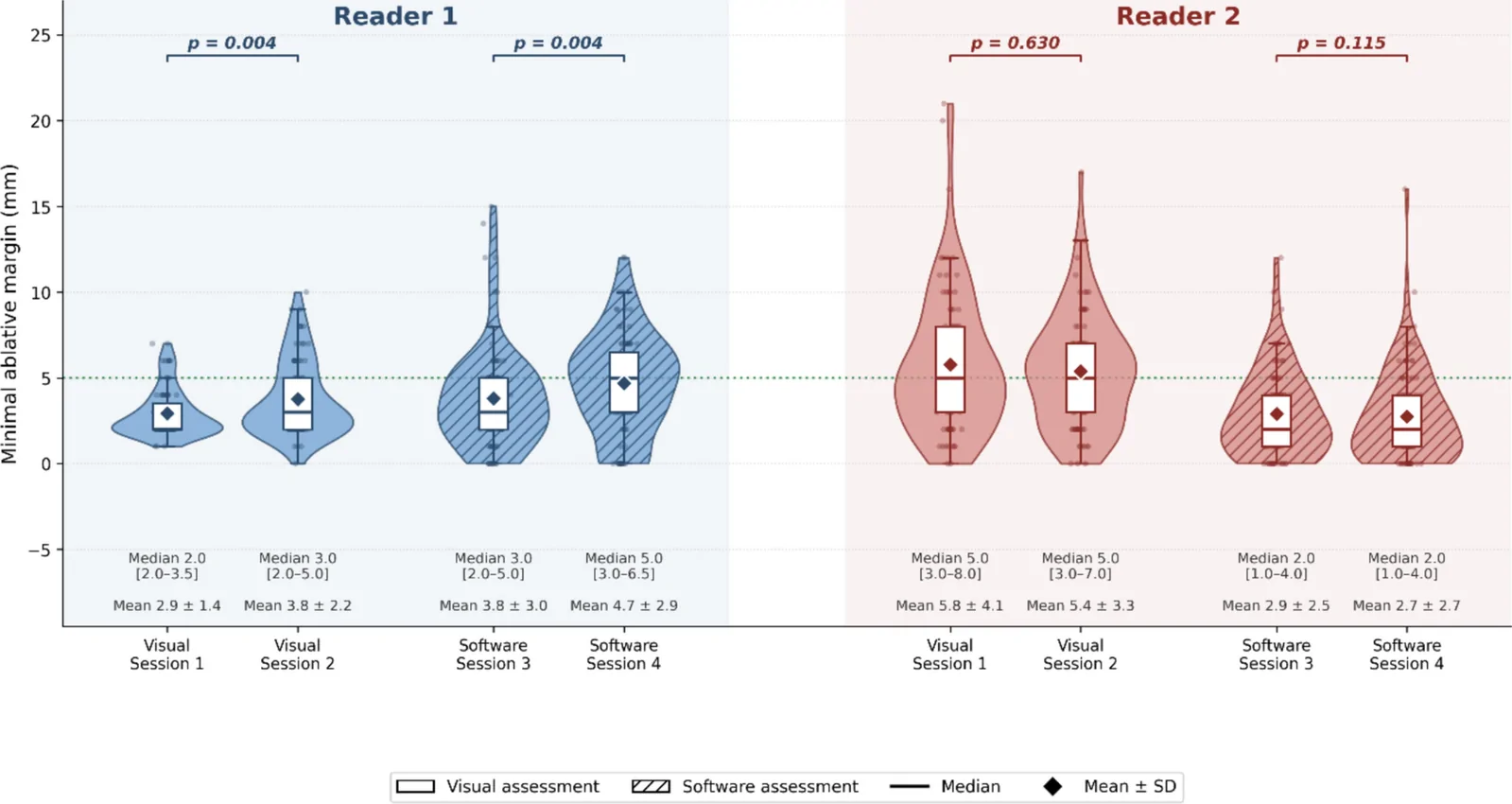
Minimal ablative margin distribution: visual versus software-assisted assessment. Each violin plot represents the distribution of MAM values across all ablated tumours (n = 87) for a given reader (Reader 1, blue; Reader 2, red), assessment method (visual, solid fill; software-assisted, hatched fill), and reading session. The white box indicates the interquartile range (25th–75th percentiles), the horizontal line within the box represents the median, and the black diamond shows the mean. Individual lesion-level MAM values are displayed as jittered dots. The green dotted line marks the 5 mm clinical threshold commonly used to define a sufficient ablative margin. Median [IQR] and mean ± standard deviation are reported below each violin. *P* values were derived from paired Wilcoxon signed-rank tests comparing session 1 versus session 2 within each reader–method combination, assessing intrareader repeatability

For reader 1, visual assessment yielded median margins of 2.0 [IQR 2.0–3.5] mm (session 1) and 3.0 [IQR 2.0–5.0] mm (session 2; *p* = 0.004), while software-assisted assessment yielded 3.0 [IQR 2.0–5.0] mm (session 3) and 5.0 [IQR 3.0–6.5] mm (session 4; *p* = 0.004). For reader 2, visual margins were 5.0 [IQR 3.0–8.0] mm (session 1) and 5.0 [IQR 3.0–7.0] mm (session 2; *p* = 0.630), whereas software-assisted values were 2.0 [IQR 1.0–4.0] mm at both sessions (*p* = 0.115) (Fig. 3).

### Intrareader Repeatability

**Fig. 4:**
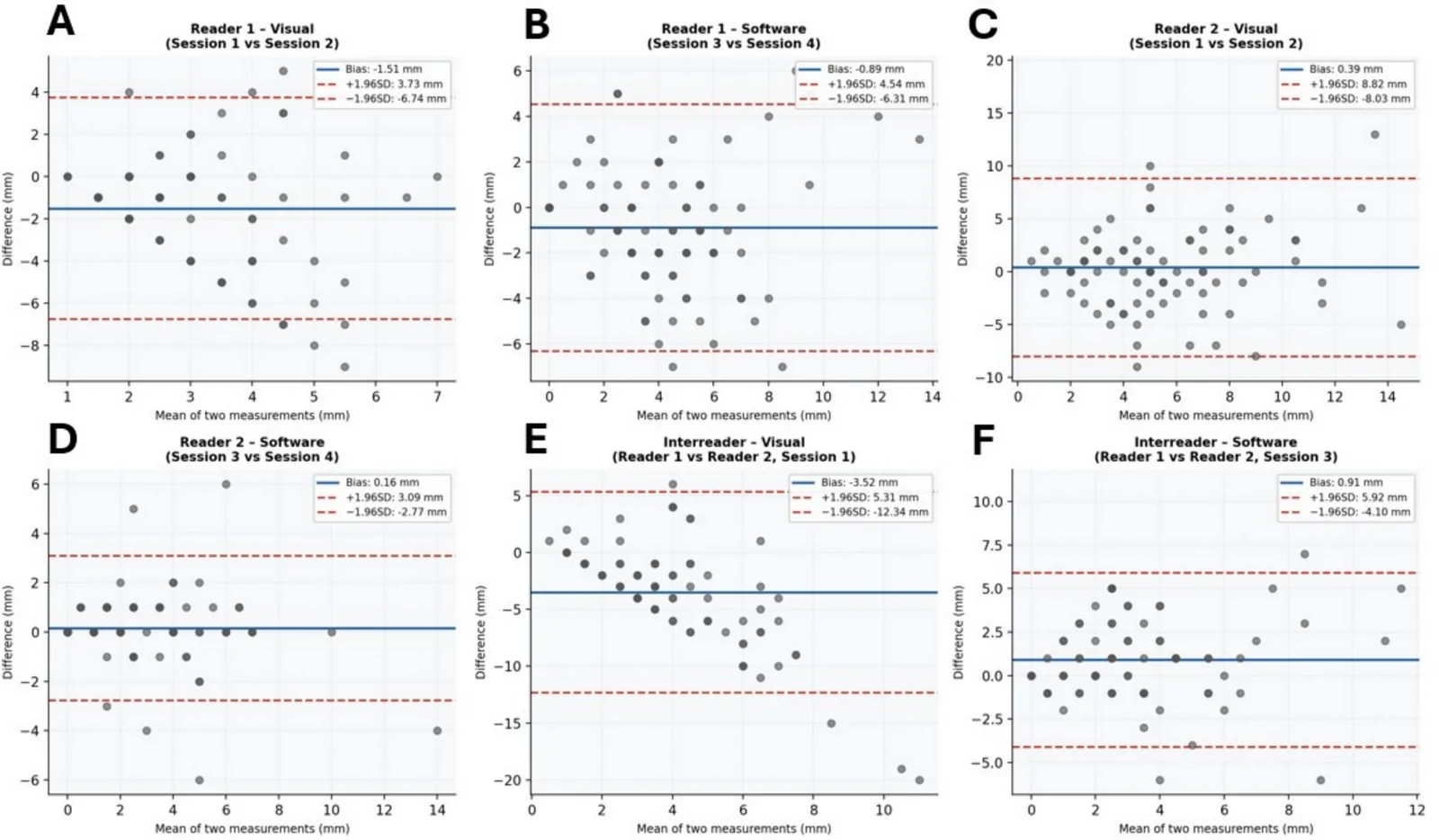
Bland–Altman analysis of minimal ablation margin. Bland–Altman plots comparing intraobserver agreement for reader 1 (**A**: visual; **B**: software) and reader 2 (**C**: visual; **D**: software), and interreader agreement (**E**: visual; **F**: software). Solid blue line: mean bias; dashed red lines: 95% limits of agreement (mean ± 1.96 SD)

For reader 1, mean bias was −0.83 mm (95%CI: −1.35, −0.31) between session 1 and session 2 and −0.89 mm between session 3 and session 4 (95%CI: −1.47, −0.30). Median absolute differences were 1.0 mm and 2.0 mm, respectively (*p* = 0.097). ICC was 0.10 [95%CI −0.11–0.31] for visual assessment and 0.54 [95%CI 0.40–0.69] for software-assisted assessment. For reader 2, mean bias was +0.39 mm (95%CI: −0.53, +1.31) between session 1 and session 2, and +0.16 mm (95%CI: −0.16, +0.48) between session 3 and session 4 (Fig. 3). Median absolute differences were 2.0 and 1.0 mm, respectively (*p* < 0.001). ICC was 0.33 [95%CI 0.12–0.50] for visual assessment and 0.83 [95%CI 0.76–0.89] for software-assisted assessment. After categorising margins into three clinically relevant groups (< 5 mm, 5–10 mm, > 10 mm), agreement between sessions 1 and 2 was κ = 0.013 [95%CI −0.15–0.21] (slight) for reader 1 and κ = 0.236 [95%CI 0.07–0.41] (fair) for reader 2. Agreement between sessions 3 and 4 improved to κ = 0.21 [95%CI 0.05–0.37] (fair) for reader 1 and κ = 0.732 [95%CI 0.49–0.92] (substantial) for reader 2 (Table 2, Fig. 4).

**Table 2:** Repeatability and reproducibility results: visual versus software-assisted assessment

Comparison	Method	ICC (95% CI)	Cohen’s κ (95% CI)	Bias, mm [95% CI]	LoA (mm)	Within ± 1 mm, n/N (%)	Within ± 2 mm, n/N (%)	Within ± 5 mm, n/N (%)	Disagreement across 5 mm, n/N (%)
Intrareader – Reader 1	Visual	0.10 [− 0.11–0.31]	0.01 [− 0.15–0.21]	− 0.83 [− 1.35– − 0.31]	[− 5.62, + 3.97]	50/87 (57)	61/87 (70)	83/87 (95)	29/87 (33)
	Software	0.54 [0.40–0.69]	0.21 [0.05–0.37]	− 0.89 [− 1.47– − 0.30]	[− 6.31, + 4.54]	35/87 (40)	56/87 (64)	82/87 (94)	30/87 (34)
Intrareader – Reader 2	Visual	0.33 [0.12–0.50]	0.24 [0.07–0.41]	+ 0.39 [− 0.53– + 1.31]	[− 8.03, + 8.82]	33/87 (38)	46/87 (53)	74/87 (85)	29/87 (33)
	Software	0.83 [0.76–0.89]	0.73 [0.49–0.92]	+ 0.16 [− 0.16– + 0.48]	[− 2.77, + 3.09]	75/87 (86)	81/87 (93)	85/87 (98)	12/87 (14)
Interreader	Visual	− 0.05 [− 0.28–0.14]	− 0.10 [− 0.20–0.02]	− 2.84 [− 3.79– − 1.89]	[− 11.55, + 5.87]	27/87 (31)	37/87 (43)	64/87 (74)	53/87 (61)
	Software	0.54 [0.41–0.70]	0.38 [0.15–0.60]	+ 0.91 [+ 0.36– + 1.45]	[− 4.10, + 5.92]	52/87 (60)	64/87 (74)	83/87 (95)	22/87 (25)

Reproducibility was assessed for each comparison (intrareader reader 1, intrareader reader 2 and interreader) using complementary metrics. The intraclass correlation coefficient (ICC; two-way mixed-effects model, absolute agreement) quantifies continuous agreement between repeated measurements. Cohen’s weighted kappa (κ) reflects categorical agreement after classifying the minimal ablative margin (MAM) into three groups: < 5 mm, 5–10 mm and > 10 mm. Bland–Altman analysis reports the mean bias (first measurement minus second) with 95% confidence interval and the 95% limits of agreement (LoA = mean bias ± 1.96 SD). The four rightmost columns report clinically interpretable agreement, expressed as the number of tumours over the total assessed (n/N) with the corresponding percentage in parentheses: the proportion of paired measurements differing by ≤ 1 mm, ≤ 2 mm and ≤ 5 mm, and the proportion of pairs in which the two measurements fell on opposite sides of the 5 mm adequacy threshold. For intrareader rows, pairs are the two repeated sessions of the same reader; for the interreader rows, pairs are the two readers (visual, session 1; software, session 3). All comparisons are based on 87 tumours

ICC, intraclass correlation coefficient; κ, Cohen’s weighted kappa; LoA, limits of agreement; MAM, minimal ablative margin; SD, standard deviation; n/N, number of tumours/total tumours assessed

### Interreader Reproducibility

Interreader agreement with visual assessment was negligible, with near-chance categorical concordance (κ =  − 0.10 [95%CI − 0.20–0.02]), a negative ICC (− 0.052 [95%CI − 0.28–0.14]), and limits of agreement spanning nearly 17 mm [− 11.55, + 5.87]. Software-assisted assessment substantially improved all metrics: ICC increased to 0.54 [95%CI 0.41–0.70], κ to 0.380 [95%CI 0.15–0.60], mean bias decreased from − 2.84 to + 0.91 mm [95%CI 0.36–1.45], and limits of agreement narrowed to [− 4.10, + 5.92] mm (Table 2). The median absolute interreader difference fell from 3.0 to 1.0 mm (both *p* < 0.001).

### Survival Analysis

Over a median imaging follow-up of 12.4 [IQR 5.7–24.6] months, 11/87 tumours (13%) developed LTP. The 1-, 2- and 3-year LTPFS per tumour were 92.9%, 85.7% and 71.6%, respectively, while corresponding OS rates were 94.1%, 88.5% and 83.3%. Distant intrahepatic and extrahepatic progression occurred in 36/67 (54%) and 33/67 (49%) patients, respectively.

### Exploratory Evaluation of Clinical Impact

**Fig. 5:**
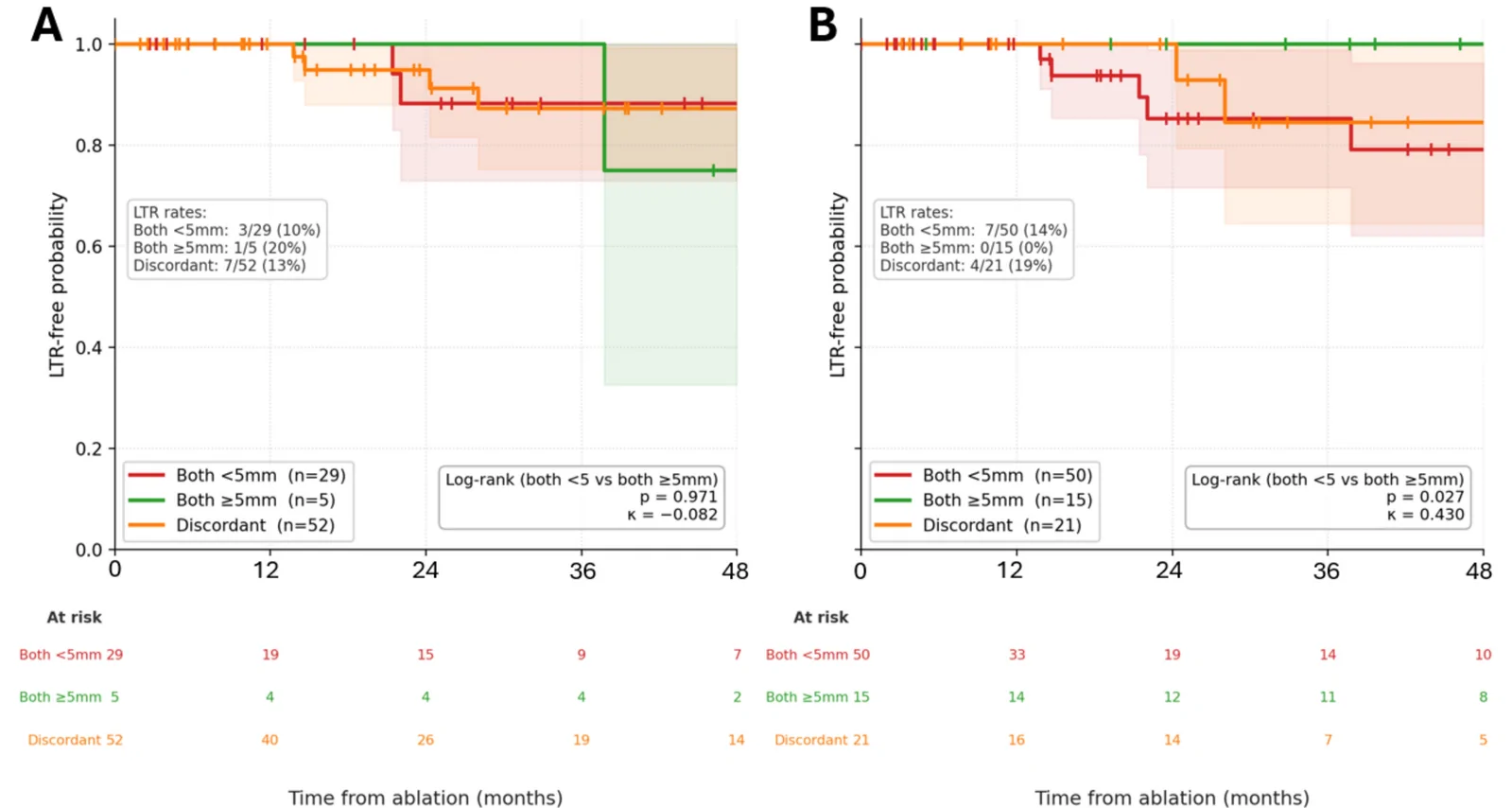
Exploratory evaluation of LTPFS according to interreader concordance on minimal ablative margin adequacy. Kaplan–Meier curves stratified by interreader concordance on the 5 mm MAM threshold (single session per method). Tumours were classified as concordant inadequate (both readers MAM < 5 mm), concordant adequate (both readers MAM ≥ 5 mm), or discordant. **A** Visual assessment (session 1): poor interreader agreement (κ =  − 0.082), 60% discordance, no significant difference in LTPFS (log-rank *p* = 0.971). **B** Software-assisted assessment (session 3): improved agreement (κ = 0.430), 24% discordance; no local progression was observed in the concordance adequate-margin group (0/15), versus 14% in the inadequate-margin group (7/50; log-rank *p* = 0.027). Tick marks: censored observations. MAM: minimal ablative margin; LTPFS: local tumour progression-free survival

With visual assessment, 60% of tumours (52/87) yielded discordant classifications between readers, and only 6% (5/87) reached concordance on an adequate margin (≥ 5 mm). Software-assisted assessment reduced the discordance rate to 24% (21/87) and identified a concordant adequate-margin group of 17% (15/87). Importantly, no LTP was observed among the 15 tumours with software-confirmed bilateral adequate margins (MAM ≥ 5 mm by both readers), compared with a 14% local progression rate in the concordant inadequate-margin group (7/50) (log-rank *p* = 0.027) (Fig. 5).

## Discussion

The principal finding of this study is that software-assisted MAM assessment improves both intra- and interreader agreement compared with visual evaluation, although interreader agreement remains only fair-to-moderate. Visual assessment showed negligible interreader agreement (κ =  − 0.10; ICC =  − 0.05), with limits of agreement approaching 17 mm, questioning its reliability for margin-based clinical decision-making. In contrast, software-assisted analysis improved interreader agreement to a fair-to-moderate level (κ = 0.38; ICC = 0.54) and reduced the limits of agreement to − 4.10 to + 5.92 mm, corresponding to approximately 40% decrease in variability. These findings are consistent with prior reports of poor reliability of visual assessment [18], and further extend the conclusions of the recent COVER-ALL randomised trial, which reported superior software-based margin evaluation during CT-guided thermal ablation [12]. The present study provides a direct comparison of visual and software-assisted margin assessment, focusing on reader reproducibility in CT-guided liver microwave ablation.

Although ablative margins are strongly associated with local tumour control, our results suggest that visual assessment may be insufficiently reliable for margin-based decision-making [3–5, 7].

Software-assisted analysis addresses several technical limitations inherent to visual evaluation, including respiratory motion, liver deformation and differences in acquisition phases. By enabling semi-automated 3D co-registration, it provides a more objective and reproducible framework for margin quantification [8–10, 19]. Nevertheless, the improvement observed in the present study was not uniform across readers, suggesting that operator experience and familiarity with the software remain relevant factors. The lack of standardisation across platforms is also a recognised issue, with significant variability in MAM values depending on the software used, highlighting the need for harmonisation before MAM can serve as a robust imaging biomarker [20, 21].

Although software-assisted assessment outperformed visual evaluation, interreader agreement reached only a fair-to-moderate level (ICC = 0.54; κ = 0.38), with limits of agreement of − 4.10 to + 5.92 mm. Several factors may explain this variability, including threshold effects around the 5 mm cut-off, reader-dependent modifications of semi-automatic tumour and ablation-zone segmentations, and the inability of rigid registration to account for non-rigid liver deformation. In addition, variability in experience and familiarity with software-assisted workflows may have contributed to the observed differences between readers. Software-assisted MAM assessment should therefore be regarded as substantially more reliable than visual assessment, but not yet sufficiently precise to be considered standardised. This residual measurement variability may also have diluted any association between MAM and LTP in the present cohort [3–5, 22, 23]. Future work should validate measurement reproducibility across software platforms, including open-source solutions recently shown to be feasible in clinical practice, and explore deformable registration to address residual variability [24, 25].

This study has several limitations. Its retrospective, single-centre design may limit generalisability. No ground truth for margin measurement was available, restricting the analysis to reproducibility rather than accuracy, while the rigid registration approach does not account for non-rigid liver deformation and may therefore introduce measurement uncertainty. The absence of LTP in the software-confirmed adequate-margin subgroup should be considered exploratory because of the small sample size, event number and follow-up duration. Finally, because software-assisted interreader agreement remained only fair-to-moderate, these findings cannot be interpreted as evidence of clinical risk stratification and require confirmation in larger prospective studies.

## Conclusion

Software-assisted ablation margin assessment improves both intrareader repeatability and interreader reproducibility compared with visual evaluation. However, interreader agreement remains only fair-to-moderate and falls short of standardisation. These findings show that visual margin estimation alone is insufficient for clinical decision-making, and support the further development and validation of software-assisted quantification.

## Supplementary Information

Below is the link to the electronic supplementary material.

Supplementary file1 (DOCX 152 kb)

## Data Availability

The data supporting the findings of this study are available from the corresponding author upon reasonable request.
