# Supplementary material for: Precision of Ablation Margin Assessment After Liver Microwave Ablation: A Head-to-Head Comparison of Visual and Software-Assisted Methods
Source: Cardiovasc Intervent Radiol. 2026 Jul 3;49(10):2105–15. doi: 10.1007/s00270-026-04528-x (PMC13642391; doi:10.1007/s00270-026-04528-x)
Supplement: Supplementary file 1 [file 270_2026_4528_MOESM1_ESM.docx]

**Supplemental 1. Intra- and interreader agreement: scatter plots.**

Scatter plots comparing repeated margin measurements for intrareader agreement in reader 1 (A: visual; B: software-assisted) and reader 2 (C: visual; D: software-assisted), and for interreader agreement (E: visual; F: software-assisted). The dashed line represents perfect agreement, and the solid line indicates the linear regression fit. Intraclass correlation coefficients (ICC) with 95% confidence intervals are reported in each panel.
